# Monoallelic *NTHL1* Loss-of-Function Variants and Risk of Polyposis and Colorectal Cancer

**DOI:** 10.1053/j.gastro.2020.08.042

**Published:** 2020-08-26

**Authors:** Fadwa A. Elsayed, Judith E. Grolleman, Abiramy Ragunathan, NTHL1 study group, Daniel D. Buchanan, Tom van Wezel, Richarda M. de Voer

**Affiliations:** 1 Department of Pathology, Leiden University Medical Center, Leiden, the Netherlands; 2 Department of Human Genetics, Radboud Institute for Molecular Life Sciences, Radboud University Medical Center, Nijmegen, the Netherlands; 3 Colorectal Oncogenomics Group, Department of Clinical Pathology, Melbourne Medical School, The University of Melbourne, Parkville, Victoria, Australia; 4 University of Melbourne Centre for Cancer Research, Victorian Comprehensive Cancer Centre, Parkville, Victoria, Australia; 5 Genomic Medicine and Family Cancer Clinic, Royal Melbourne Hospital, Parkville, Victoria, Australia

**Keywords:** Colorectal Cancer, Base Excision Repair, Tumor Mutational Signatures, Mutation Carrier

The endonuclease III-like protein 1, encoded by *NTHL1*, is a bifunctional glycosylase involved in base-excision repair (BER) that recognizes and removes oxidized pyrimidines.^1^ Similar to biallelic loss-of-function (LoF) variants in *MUTYH*,^2^ biallelic LoF variants in *NTHL1* predispose to colorectal polyps and colorectal cancer (CRC).^3^ Recently, a multitumor phenotype was observed in individuals diagnosed with NTHL1 deficiency.^4^ Carriers of monoallelic pathogenic variants in *MUTYH* have an increased, albeit small, risk of CRC.^5^ Thus far, it is unknown if monoallelic *NTHL1* LoF variants also increase the risk of polyposis and/or CRC. This information is especially important for carriers of the most common LoF variant in *NTHL1* (p.(Gln90*); NM_002528.5), which is heterozygous in approximately 0.28% of the general population.^6^ Identification of monoallelic *NTHL1* LoF variants currently presents a clinical conundrum regarding how best to counsel carriers with respect to their cancer risk because of the lack of published evidence. Here, we show that monoallelic LoF variants in *NTHL1* are not enriched in individuals with polyposis and/or CRC compared to the general population. Furthermore, 13 colorectal tumors from *NTHL1* LoF carriers did not show a somatic second hit, and we did not find evidence of a main contribution of mutational signature SBS30, the signature associated with NTHL1 deficiency, suggesting that monoallelic loss of *NTHL1* does not substantially contribute to colorectal tumor development.

## Methods

A total of 5,942 individuals with unexplained polyposis, familial CRC, or sporadic CRC at young age or suspected of having Lynch syndrome with CRC or multiple adenomas were included in this study and defined as case patients (individual studies and their ascertainment are described in Supplementary Methods and Supplementary Table 1). Three independent data sets were used as controls, including (1) the non-Finnish European subpopulation of the genome aggregation database (gnomAD: n = 64,328),^6^ (2) a Dutch cohort of individuals without a suspicion of hereditary cancer who underwent whole-exome sequencing (WES) (Dutch WES; n = 2,329),^7^ and (3) a population-based and cancer-unaffected cohort from the Colon Cancer Family Registry Cohort (CCFRC; n = 1,207) (Supplementary Methods and Supplementary Table 1).

Pathogenic *NTHL1* LoF variants were identified in case patients by sequencing the exonic regions of *NTHL1* (n = 3,439) or by genotyping of 2 LoF variants in *NTHL1* (c.268C>T, p.(Gln90*); n = 2503 and c.806G>A, p.(Trp269*); n = 261) (Supplementary Table 1). For control individuals, all pathogenic LoF variants were retrieved from gnomAD and the Dutch WES-cohort,^6,7^ and for the CCFRC control individuals, the exonic regions of *NTHL1* were sequenced (Supplementary Table 1). Odds ratios between case patients and control groups were calculated and a Fisher exact test was performed to assess the significance of difference in carrier rates. Cosegregation analysis was performed by using Sanger sequencing. Two adenomas and 11 primary CRCs from *NTHL1* LoF variant carriers were subjected to WES, and subsequently, mutational signature analysis was performed (Supplementary Methods and Supplementary Table 2). For signature analysis comparison, we included 3 CRCs from individuals with a biallelic *NTHL1* LoF variant.

## Results

**Figure 1.**
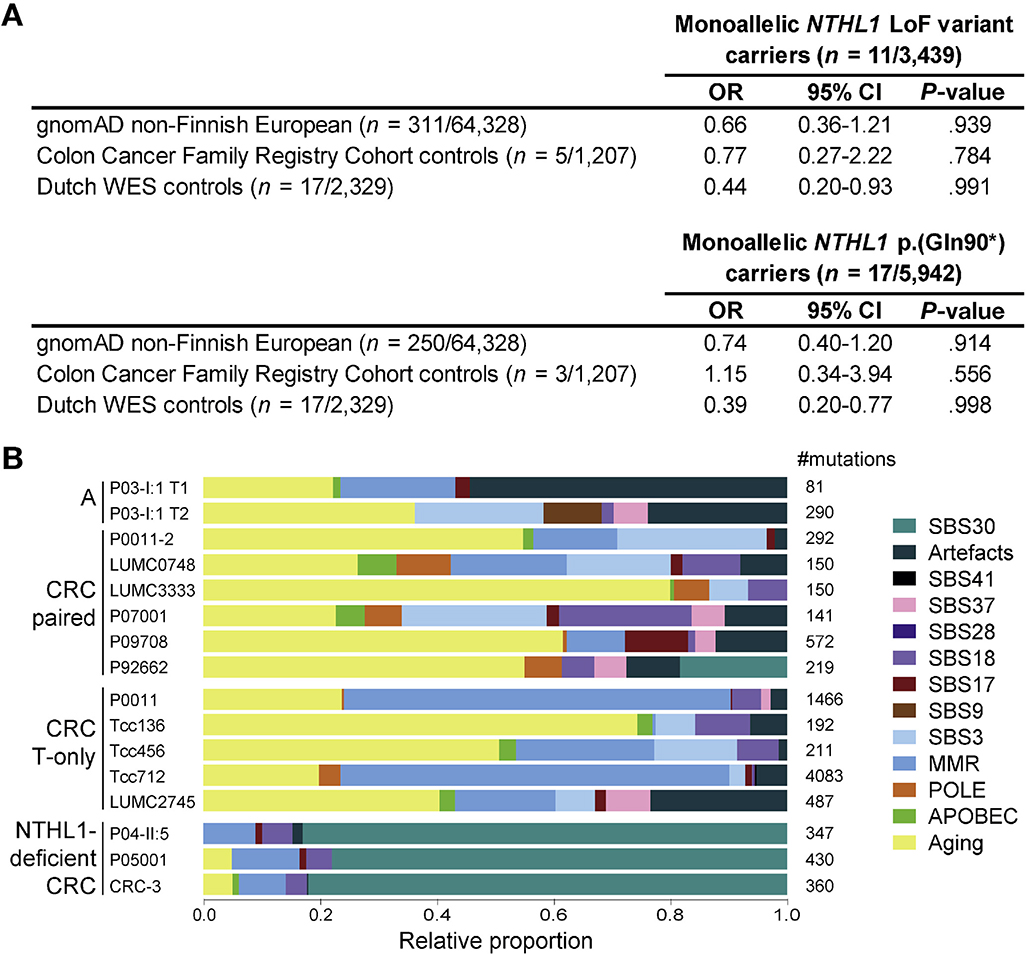
Enrichment and mutational signature analysis of *NTHL1* LoF variants in individuals with polyposis and/or CRC (case patients). (*A*) Frequencies of germline monoallelic *NTHL1* LoF variants and monoallelic *NTHL1* p.(Gln90*) variants in individuals with polyposis and/or CRC (case patients) compared with control populations. (B) Mutational signature analysis of tumors from carriers with a monoallelic *NTHL1* LoF variant. Mutational signatures with shared etiologies were grouped for display purposes, which are the signatures associated with aging (SBS1, SBS5, and SBS40), DNA mismatch repair deficiency (SBS6, SBS15, SBS20, SBS21, SBS26, and SBS44), Polymerase Epsilon (POLE) exonuclease domain deficiency (SBS10a and SBS10b), Apolipoprotein B mRNA editing enzyme (APOBEC) activity (SBS2 and SBS13), and artifact signatures (SBS45, SBS51, SBS52, SBS54, and SBS58). Data availability: paired: tumor and normal or tumor data were available; T-only: only data from 1 tumor tissue were available. A, adenomatous polyp; CI, confidence interval; OR, odds ratio.

Monoallelic *NTHL1* LoF variants were identified in 11 of 3,439 case patients (0.32%) and in 5 of 1,207 (0.41%) of CCFRC control individuals, indicating no significant difference (*P* = .784) (Figure 1A, Supplementary Table 1). Genotyping of the *NTHL1* p.(Gln90*) variant in another 2,503 case patients identified 7 additional carriers (0.28%). The overall frequency of *NTHL1* p.(Gln90*) in case patients was not different from the frequency in the gnomAD (17/5,942 vs 250/64,328; *P* = .914), CCFRC (17/5,942 vs 3/1,207; *P* = .556) or Dutch WES control individuals (17/5,942; vs 17/2,329; *P* = .998) (Figure 1A and Supplementary Table 1).

Via cosegregation analysis, we identified 3 additional *NTHL1* p.(Gln90*) carriers. The phenotype of all carriers identified in this study is described in Supplementary Table 2. Thirteen colorectal tumors from *NTHL1* LoF carriers underwent WES (details in Supplementary Table 2). The *NTHL1* wild-type allele was unaffected by somatic mutations or loss of heterozygosity in all tumors tested. In contrast to *NTHL1*-deficient tumors, in none of the tumors of the carriers was mutational signature SBS30 the main signature, because it was only present in 1 tumor, where it had a minor contribution (Figure 1B and Supplementary Table 2).^4^ These observations indicate that biallelic inactivation of *NTHL1* through a somatic second hit was not evident and that monoallelic inactivation of *NTHL1* was insufficient to result in the accumulation of somatic mutations that are characteristic of an *NTHL1*-deficiency phenotype.

## Discussion

In this study, the largest investigating monoallelic LoF variants in *NTHL1* to date to our knowledge, we observed no evidence of an association between carriers and the risk of polyposis and/or CRC. In our case patients, the prevalence of pathogenic *NTHL1* LoF variant alleles is comparable to that of the general population. However, we cannot rule out that a small risk for CRC, similar to what is observed for *MUTYH* carriers, still exists.

Colorectal tumors from monoallelic *NTHL1* LoF variant carriers did not show evidence of a somatic second hit in *NTHL1* nor of defective base-excision repair, which is typically associated with biallelic *NTHL1* inactivation. Only 1 tumor showed a minor SBS30 contribution to the mutation profile, but this contribution was far less significant compared to NTHL1-deficient CRC and is likely the result of multiple testing correction. Our data suggest that inactivation of the *NTHL1* wild-type allele is a rare event in colorectal tumors, which is in agreement with the observation that loss of heterozygosity of chromosome arm 16p is not frequently observed in CRC.^8^ We were unable to discriminate between individuals with polyposis or CRC due to the historical nature of the case collections. Therefore, differences in the frequencies of monoallelic *NTHL1* LoF variants between control individuals and these 2 phenotypes were not made separately. However, because we identified *NTHL1* LoF variants in individuals with polyposis or CRC, we do not consider a major difference between these 2 phenotypes. Because NTHL1 deficiency may also predispose to extracolonic tumors, the risk for these tumor types in monoallelic *NTHL1* carriers still needs further assessment.

In conclusion, the evidence to date does not support an increased risk of polyposis and/or CRC for carriers of monoallelic *NTHL1* LoF variants, and consequently, no additional surveillance is currently warranted beyond population screening for CRC, unless family history characteristics point to a reason for colonoscopy.

## Supplementary Material

1

## Abbreviations used in this paper:

CCFRC: Colon Cancer Family Registry Cohort
CRC: colorectal cancer
LoF: loss of function
WES: whole-exome sequencing

## References

[1] Krokan HE, Bjørås M. Cold Spring Harb Perspect Biol 2013;5:a012583. 23545420 10.1101/cshperspect.a012583 PMC3683898

[2] Al-Tassan N, Nat Genet 2002;30:227–232. 11818965 10.1038/ng828

[3] Weren RD, Nat Genet 2015;47:668–671. 25938944 10.1038/ng.3287

[4] Grolleman JE, de Voer RM, Elsayed FA, Cancer Cell 2019;35:256–266. 30753826 10.1016/j.ccell.2018.12.011

[5] Win AK, Int J Cancer 2011;129:2256–2262. 21171015 10.1002/ijc.25870 PMC3291738

[6] Karczewski KJ, Nature 2020;581:434–443. 32461654 10.1038/s41586-020-2308-7 PMC7334197

[7] de Voer RM, Hahn MM, Mensenkamp AR, Sci Rep 2015;5:14060. 26358404 10.1038/srep14060 PMC4566092

[8] Cerami E, Cancer Discov 2012;2:401–404. 22588877 10.1158/2159-8290.CD-12-0095 PMC3956037

