# Supplementary material for: Monoallelic NTHL1 Loss-of-Function Variants and Risk of Polyposis and Colorectal Cancer
Source: Gastroenterology. Author manuscript; Available in PMC 2021 Feb 22. (PMC7899696; doi:10.1053/j.gastro.2020.08.042)
Supplement: 1 [file NIHMS1658701-supplement-1.pdf]

Supplementary Methods

Study Cohorts

We included 5,942 patients with unexplained polyposis, familial CRC, or sporadic CRC at a young age or suspected of having Lynch syndrome with CRC or multiple adenomas (Supplementary Table 1) from the Netherlands (n = 3,158); United Kingdom (n = 275); Poland (n = 144); Germany (n = 104); Spain (n = 35); North Macedonia (n = 273); and North America, Canada, and Australia (CCFRC; n = 1,953).[1-3] All participants provided written informed consent. Local medical ethical committees approved this study (Radboudumc [Commissie mensgebonden onderzoek (CMO)-light, 2015/2172 and 2015/1748], Leiden University Medical Center (LUMC) [P01-019], and Ontario Cancer Research Ethics Board, University of Melbourne Human Research Ethics Committee, and Fred Hutchinson Cancer Research Center institutional review board).

A total of 1,207 cancer-unaffected control individuals were available from the population-based recruitment arms of the CCFRC.[2,3] From the Netherlands, 2,329 WES control individuals with a >90-fold median coverage without a suspicion of hereditary cancer were available.[4] The European non-Finnish population of gnomAD was used to determine overall frequencies of LoF variants.[5]

Targeted Resequencing

Hi-Plex. Leukocyte DNA from 1,953 CRC-affected case patients and 1,207 control individuals was used to screen the coding regions of *NTHL1* by using multiplex polymerase chain reaction (PCR)–based targeted sequencing and variant calling approach (HiPlex2 and Hiplexpipe, hiplex.org, github.com/khalidm/hiplexpipe).[6] Germline variants in *NTHL1* (NM_002528.5) were prioritized according to quality—the sequence depth of >30 reads and variant frequency of >30%.

Molecular Inversion Probe–Based Sequencing. Leukocyte DNA from 1,486 polyposis and/or CRC cases was screened for all coding regions and intron–exon boundaries of *NTHL1* (NM_002528.5) by using molecular inversion probe MIPsequencing, combined with a panel of base excision repair genes, as described previously.[1] Reads were mapped with Burrows-Wheeler Aligner (BWA), and variant calling was performed with UnifiedGenotyper.[7] Somatic variants in *NTHL1* were prioritized according to quality: sequence depth of >40 reads, >20 variant reads, variant frequency of >25%, and quality by depth scores >8,000.

Variants from HiPlex and MIP screenings were further selected based on predicted LoF of *NTHL1*. We selected all nonsense, frameshift canonical splice sites and included only coding and noncoding splice site region variants with a predicted change of >20%, based on Alamut (Interactive Biosoftware, Rouen, France) (MaxEnt, NNSplice, and Human Splicesite Finder [HSF]).

KASPar Assay

Leukocyte DNA (n = 1,260) or germline DNA extracted from formalin-fixed, paraffin embedded (FFPE) surgical specimens (n = 982) was genotyped for *NTHL1* p.(Gln90*) by using KBioscience Competitive Allele-Specific PCR (KASPar) assay.[1]

Allele-Specific Polymerase Chain Reaction

Leukocyte DNA from 261 individuals with sporadic or familial CRC was subjected to an allele-specific PCR (AS-PCR) specific for *NTHL1* p.(Gln90*) and p.(Trp269*); primers are available upon request.

Sanger Sequencing

Sanger sequencing was used for variant validation and to sequence the entire open reading frame of *NTHL1* in confirmed heterozygous cases. In addition, when available, family members were sequenced by using Sanger sequencing for cosegregation purposes.

Statistical analysis

A 1-sided Fisher exact test was performed to determine differences in the frequency of monoallelic *NTHL1* germline LoF variants in carriers with polyposis and/or CRC compared to control individuals. We calculated the *P* value, odds ratio, and the 95% confidence interval using R (R Foundation for Statistical Computing, Vienna, Austria; http://www.R-project.org). Three control data sets were used in this comparison.

First, we retrieved all LoF variants (nonsense, frameshift canonical splice sites, and coding or noncoding splice site regions with >20% splice site change) in canonical transcripts of *NTHL1* listed in the non-Finnish European subpopulation of the genome aggregation database (gnomAD).[5] All variants were checked manually in gnomAD for their quality. Second, LoF variants in *NTHL1* identified in the Dutch WES cohort (n = 2,329 individuals without a suspicion of hereditary cancer) were extracted in a similar way as described earlier.[4] Third, LoF variants in *NTHL1* identified in the CCFRC control group of 1,207 individuals, sequenced in this study, were used.

Whole-Exome Sequencing

Exome captures (Supplementary Table 2) were performed according to the manufacturer by using either Agilent Clinical Research Exome (CRE) V2 (Agilent, Santa Clara, CA) in combination with sequencing on a NovaSeq 6000 (Illumina, San Diego, CA), Agilent SureSelect XT^{HS} Human All Exon V6 enrichment kit in combination with sequencing on a NextSeq 500, or xGEN Exome Research Panel (Integrated DNA Technology [IDT], Coralville, IA) in combination with sequencing on a NovaSeq 6000.

Novaseq 6000 sequencing reads were trimmed by using Trimmomaticv0.36 and aligned to hs37d5 by using BWA-MEM, followed by merging and PCR duplicate removal with Sambamba (version 0.5.8).[8,9] Variant calling was performed bt using Strelka (version 2.017) and Freebayes for paired samples; only variants called by both callers were reported.[10,11] For LUMC2745, no paired sample was available, and variant calling was performed with Mutect2 (GATK version 4.1.0.0; GATK, Broadinstitute, Cambridge, MA).

Trimmed NextSeq 500 sequencing reads were aligned to GRCh37 by using BWA-MEM, and duplicates were flagged by using Picard Tools, version 1.90. Variants were called with Mutect2 (GATK version 4.1.0.0), with or without matched germline samples; variant filtering was performed as described,[1] with minor modifications. Variants in dbSNPv132 (minus catalogue of somatic mutations in cancer [COSMIC]), microsatellites, homopolymers, simple repeats, and variants called outside of the respective exome capture target were removed. Somatic variants with a variant allele frequency of $<10\%$, $<20\times$ coverage in both normal and tumor, and fewer than 4 reads supporting the variant were removed. For tumor-only analysis, variants shared by more than 1 individual and variants with a variant allele frequency of $>80\%$ were removed to reduce germline leakage.

Mutational Signature Analysis

Mutation spectra were generated by using In-depth characterization and analysis of mutational signatures (ICAMS), version 2.1.2 (github.com/steverozen/ICAMS), and mutational signature analysis was performed by using mSigAct v2.0.0.9018.[12] Tissue-specific CRC signature universes were inferred from the Pan-cancer analysis of whole genomes (PCAWG) signature assignments.[13] The signature universe was extended with SBS30 and potential artefact signatures SBS45, SBS51, SBS52, SBS54, and SBS58, which were present in a subset of the samples of this cohort. Signatures were normalized to the trinucleotide abundance of the respective exome capture panel used. Per mutation spectrum, mutational signature assignment was performed by using mSigAct::SparseAssignActivity, with $P = .5$ to reduce sparsity. The presence of SBS30 was then determined using mSigAct::SignaturePresenceTest using the signatures determined by mSigAct::SparseAssignActivity plus SBS30 as well as the aging-associated signatures SBS1, SBS5, and SBS40 (Supplementary Table 2). Multiple testing correction was done according to Benjamini-Hochberg.

References

1. **Grolleman JE, de Voer RM, Elsayed FA**, et al. Mutational signature analysis reveals NTHL1 deficiency to cause a multi-tumor phenotype. Cancer Cell 2019; 35:256–266.
2. Jenkins MA, Win AK, Templeton AS, et al. Cohort Profile: The Colon Cancer Family Registry Cohort (CCFRC). Int J Epidemiol 2018;47:387–388i.
3. Newcomb PA, Baron J, Cotterchio M, et al. Colon Cancer Family Registry: an international resource for studies of the genetic epidemiology of colon cancer. Cancer Epidemiol Biomarkers Prev 2007;16:2331–2343.
4. **de Voer RM, Hahn MM, Mensenkamp AR**, et al. Deleterious germline BLM mutations and the risk for early-onset colorectal cancer. Sci Rep 2015;5:14060.
5. Karczewski KJ, Francioli LC, Tiao G, et al. The mutational constraint spectrum quantified from variation in 141,456 humans. Nature 2020;581:434–443.
6. Hammet F, Mahmood K, Green TR, et al. Hi-Plex2: a simple and robust approach to targeted sequencing-based genetic screening. Biotechniques 2019;67:118–122.
7. DePristo MA, Banks E, Poplin R, et al. A framework for variation discovery and genotyping using next-generation DNA sequencing data. Nat Genet 2011;43:491–498.
8. Bolger AM, Lohse M, Usadel B. Trimmomatic: a flexible trimmer for Illumina sequence data. Bioinformatics 2014;30:2114–2120.
9. Tarasov A, Vilella AJ, Cuppen E, et al. Sambamba: fast processing of NGS alignment formats. Bioinformatics 2015;31:2032–2034.
10. Garrison E, Marth G. Haplotype-based variant detection from short-read sequencing. arXiv; 2012. Available at: https://arxiv.org/abs/1207.3907v2 Accessed October 25, 2020.
11. Saunders CT, Wong WS, Swamy S, et al. Strelka: accurate somatic small-variant calling from sequenced tumor-normal sample pairs. Bioinformatics 2012;28:1811–1817.
12. **Ng AWT, Poon SL**, Huang MN, et al. Aristolochic acids and their derivatives are widely implicated in liver cancers in Taiwan and throughout Asia. Sci Transl Med 2017;9(412):eaan6446.
13. **Alexandrov LB, Kim J, Haradhvala NJ**, et al. The repertoire of mutational signatures in human cancer. Nature 2020;578:94–101.
14. Vos JR Manders P, de Voer RM, et al. Parelsnoer Institute Biobank Hereditary Colorectal Cancer: a joint infrastructure for patient data and biomaterial on hereditary colorectal cancer in the Netherlands. Open J Bioresources 2019;6,:1; Doi: http://doi.org/10.5334/ojb.54.

Author names in bold designate shared coirst authorship.

Supplementary Table 1. Characteristics of Case and Control Cohorts and Identified Case Patients and Control Individuals With Monoallelic *NTHL1* LoF Variants in This Study

Approach	Sequencing method and cohorts	Samples, n	Selection[a] criteria	Genes tested	Monoallelic NTHL1 p.(Gln90*), n	Other monoallelic NTHL1 LoF variants, n	Total monoallelic NTHL1 LoF variants, n
NTHL1-targeted resequencing (n = 3,439 cases)	Hi-Plex multiplex PCR-based sequence screening of NTHL1 exons (control individuals)						
	Colon Cancer Family Registry	1,207	Population-based healthy individuals with no history of polyposis and/or CRC	NA	3	2	5
	Hi-Plex multiplex PCR based sequence screening of NTHL1 exons (case patient)						
	Colon Cancer Family Registry	1,953	Population-based CRC	*APC*, *MUTYH*, *POLE*, *POLD1,* MMR*	4	1	5
	MIP-based sequence screening of NTHL1 (case patients)						
	ParelBED (the Netherlands[b])	600	Polyposis, CRC, or CRC and additional tumor	No disease-causing mutation found after routine diagnostics	0	0	0
	Oxford (United Kingdom)	275	Polyposis	*APC*, *MUTYH*	4	0	4
	Leiden (the Netherlands)	150	Polyposis or familial CRC	*APC*, *MUTYH*	0	0	0
	Nijmegen (the Netherlands)	147	Polyposis or familial CRC	*APC*, *MUTYH*	0	0	0
	Szczecin (Poland)	144	Familial CRC	*POLE*, *POLD1*, MMR*[b]	1	0	1
	Dresden (Germany)	104	Polyposis or familial CRC	*APC*, *MUTYH*	0	0	0
	Santiago de Compostela (Spain)	35	Polyposis or familial CRC	*APC*, *MUTYH* (in part), *POLE*, *POLD1, BMPR1A, SMAD4, PTEN*	0	0	0
	Groningen (the Netherlands)	19	Polyposis or familial CRC	*APC*, *MUTYH*	0	0	0
	Skopje (North Macedonia)	12	Polyposis, recessive inheritance	MMR*,[b] *APC*, *TP53*, *MUTYH*, *POLE*, *POLD1*	1	0	1

Supplementary Table 1. Continued

Approach	Sequencing method and cohorts	Samples, n	Selection[a] criteria	Genes tested	Monoallelic NTHL1 p.(Gln90*), n	Other monoallelic NTHL1 LoF variants, n	Total monoallelic NTHL1 LoF variants, n
NTHL1 genotyping (*n* = 2,503 cases)	*NTHL1* p.(Gln90*) genotyping by KASPar assay (case patients)						
	Leiden (the Netherlands)	1,894	Polyposis or familial CRC, with or without suspected Lynch syndrome	*APC*, *MUTYH*, *POLE*, *POLD1,* MMR*[b]	3	NA	3
	Nijmegen (the Netherlands)	348	Polyposis or familial CRC	*APC*, *MUTYH*, *POLE*, *POLD1,* MMR*[b]	1	NA	1
	NTHL1 p.(Gln90*) and p.(Trp269*) genotyping by allele specific-PCR (case patients)						
	Skopje (North Macedonia)	200	Sporadic CRC	None	2	0	2
	Skopje (North Macedonia)	61	Polyposis or familial CRC	TruSight Hereditary Cancer Panel (Illumina)	1	0	1

NA, not applicable; ParelBED, The Dutch Parelsnoer Institute Biobank Hereditary Colorectal Cancer.[14]
[a]Polyposis is defined as the cumulative occurrence of at least 10 polyps. Familial CRC is defined as the proband having a CRC ≤50 years of age and at least 1 first degree relative with CRC ≤60 years of age. Sporadic CRC is defined as patients with CRC without a family history, irrespective of age.
[b]MMR* genes: *MLH1*, *MSH2*, MSH6 and *PMS2*.

Supplementary Table 2. Phenotypic Description and Details on the Tumors Subjected to WES of Identified Carriers of a Monoallelic *NTHL1* LoF Variant

Number	Patient ID	Identification method	Amino acid change	Sex	Polyps	Malignancies[i]	Tumor type for WGS	Matched normal available	Exome enrichment kit	Sequencing platform	Median coverage tumor(s)[j]	Number of somatic variant calls	*P* value SBS30[a]
1	P09708	Hi-Plex	p.(Gln287*)	M		Cecum (73), CRC (73)	CRC	Yes, blood	Agilent CRE V2	Novaseq 6000	221	572	.976
2	P92662	Hi-Plex	p.(Gln90*)	M		CRC (53)	CRC	Yes, blood	Agilent CRE V2	Novaseq 6000	189	219	1.61×10^{-3}
3	P07001	Hi-Plex	p.(Gln90*)	M		CRC (43)	CRC	Yes, blood	Agilent CRE V2	Novaseq 6000	116	141	.331
4	P58832	Hi-Plex	p.(Gln90*)	F		CRC (46), UC (29)	—	—	—	—	—	—	—
5	P00387	Hi-Plex	p.(Gln90*)	F		Cecum (42), UC (23), LC (53)	—	—	—	—	—	—	—
6	P0011[b]	MIP screen	p.(Gln90*)	M		CRC (56), LiC (unk)	CRC	No[c]	Agilent V6	NextSeq500	133	1,466	.976
7	P0011-2[b]	Cosegregation	p.(Gln90*)	F		CRC (55)	CRC	Yes, FFPE	Agilent V6	NextSeq500	86	292	.953
8	P0804	MIP screen	p.(Gln90*)	F		CRC (50)	CRC[d]	Yes, FFPE	Agilent V6	NextSeq500	—	—	—
9	P0468[e]	MIP screen	p.(Gln90*)	M	A (43)		—	—	—	—	—	—	—
10	P0567[e]	Co-segregation	p.(Gln90*)	F	A (55)		—	—	—	—	—	—	—
11	P0567-2[e]	Co-segregation	p.(Gln90*)	F	A (61)		—	—	—	—	—	—	—
12	P0523	MIP screen	p.(Gln90*)	M	A (59)	CRC (58)	—	—	—	—	—	—	—
13	P0568	MIP screen	p.(Gln90*)	M	A (unk)		—	—	—	—	—	—	—
14	P0602	MIP screen	p.(Gln90*)	F	A (unk)		—	—	—	—	—	—	—
15	K134	KASPar assay	p.(Gln90*)	F	A (48-56)	CRC (49)	—	—	—	—	—	—	—
16	LUMC3333	KASPar assay	p.(Gln90*)	M		CRC (<69), Cecum (69)	CRC	Yes, FFPE	IDT xGEN	Novaseq 6000	131	150	.888
17	LUMC2745	KASPar assay	p.(Gln90*)	M		CRC (72); CRC, SCC (61)	CRC	No	IDT xGEN	Novaseq 6000	99	487	.053
18	LUMC0748	KASPar assay	p.(Gln90*)	F		CRC (56), OvC (56), CRC (56), CRC (68)	CRC	Yes, FFPE	IDT xGEN	Novaseq 6000	84	150	>.99
19	Tcc136	AS-PCR	p.(Gln90*)	M		CRC (75)	CRC[f]	No	Agilent V6	NextSeq500	195	192	.331

Supplementary Table 2. Continued

Number	Patient ID	Identification method	Amino acid change	Sex	Polyps	Malignancies[i]	Tumor type for WGS	Matched normal available	Exome enrichment kit	Sequencing platform	Median coverage tumor(s)[j]	Number of somatic variant calls	*P* value SBS30[a]
20	Tcc456	AS-PCR	p.(Gln90*)	M		PC, CRC (72)	CRC	No	Agilent V6	NextSeq500	140	211	.052
21	Tcc712	AS-PCR	p.(Gln90*)	F	7A (71)	EC (66), CRC (71)	CRC[f]	No	Agilent V6	NextSeq500	180	4,083	1
22	P03-I:1	[g]	p.(Gln90*)	M		A, HP	A	No	IDT xGEN	Novaseq 6000	T1 = 64	T1 = 81	T1 = 1
											T2 = 39	T2 = 290	T2 = .088
—	P04-II:5	[g]	p.Gln90*/ p.Ile245Asnfs*28	F	—	—	NTHL1-deficient CRC	Yes, FFPE	IDT xGEN	Novaseq 6000	162	347	3.11×10^{-45}
—	P05001	Hi-Plex	p.(Gln90*)/ p.(Ala79fs)	F	A, HP (61)	CRC (61), BCC (63)	NTHL1-deficient CRC	Yes, blood	Agilent CRE V2	Novaseq 6000	108	430	1.82×10^{-39}
—	CRC-3	[h]	p.(Gln90*)/ p.(Gln90*)	M	—	—	NTHL1-deficient CRC	Grolleman et al[1]	Grolleman et al[1]	Grolleman et al[1]	Grolleman et al[1]	360	3.08×10^{-38}

A, colorectal adenomatous polyps; BCC, basal cell carcinoma; EC, endometrial cancer; HP, hyperplastic polyps; ID, identifier; LC, lung cancer; LiC, liver cancer; OvC, ovarian cancer; PC, prostate cancer; SCC, squamous cell carcinoma; UC, uterine cancer; unk, age unknown; —, not applicable.
[a]Fresh-frozen tumor material.
[b]Sibling.
[c]The normal sample of the sibling was used for somatic variant extraction.
[d]Tumor P0804 was excluded from further analysis because eof insufficient data quality.
[e]Sibling.
[f]Multiple testing correction was done according to Benjamini-Hochberg.
[g]Identified by Grolleman et al, 2019.[1]
[h]Tumor data from Grolleman et al, 2019.[1]
[i]Numbers in parentheses indicate age at diagnosis.
[j]Median read coverage (units = reads).
